# Fish gut-associated bacterial communities in a tropical lagoon (Aghien lagoon, Ivory Coast)

**DOI:** 10.3389/fmicb.2022.963456

**Published:** 2022-09-29

**Authors:** Alison Gallet, Eric Kouamé Yao, Pierre Foucault, Cécile Bernard, Catherine Quiblier, Jean-François Humbert, Julien Kalpy Coulibaly, Marc Troussellier, Benjamin Marie, Sébastien Duperron

**Affiliations:** ^1^UMR 7245 Molécules de Communication et Adaptation des Micro-Organismes, Muséum National d’Histoire Naturelle, Centre National de la Recherche Scientifique, Paris, France; ^2^Institut Pasteur de Côte d’Ivoire, Abidjan, Côte d’Ivoire; ^3^Université Paris Cité, UFR Sciences du Vivant, Paris, France; ^4^INRAE-iEES Paris, Sorbonne Université, Paris, France; ^5^MARBEC, Centre National de la Recherche Scientifique, Université Montpellier, IFREMER, IRD, Montpellier, France

**Keywords:** *Hemichromis*, *Tilapia*, *Sarotherodon*, *Chrysichthys*, Cichlidae fish, Claroteidae

## Abstract

Aghien lagoon (Ivory Coast) is a eutrophic freshwater lagoon that harbors high biomasses of phytoplankton. Despite Increasing interest in fish gut microbiomes diversity and functions, little data is currently available regarding wild species from tropical west African lakes. Here, gut-associated bacterial communities are investigated in four fish species that are consumed by locale populations, namely the Cichlidae *Hemichromis fasciatus*, *Tilapia guineensis* and *Sarotherodon melanotheron*, and the Claroteidae *Chrysichthys nigrodigitatus*. Species-related differences are identified, that can be attributed to host phylogeny and diet. Important variations throughout the year are observed in *T. guineensis* and *C. nigrodigitatus*. This result emphasized the importance of time-series sampling and comparison with environmental variables even in tropical regions, that are not often conducted in wild populations. Effects of environmental factors (anthropogenic or not) on the microbiota and potential outcomes for fish health and populations sustainability need to be further explored. Interestingly, fish appear as major reservoirs of bacterial diversity, suggesting that they could contribute to the overall stability and resilience of bacterial communities present in the Aghien lagoon.

## Introduction

The study of fish biology and ecology has embraced the microbiome revolution over the past decade (McFall-Ngai et al., 2013). Gut-associated bacterial communities have been investigated in various fish species, where they were shown to play multiple roles relevant to host physiology, nutrition, immunity, protection, and behavior (Llewellyn et al., 2014; Egerton et al., 2018). To date, most studies have focused on species of economical relevance and investigated the impact of gut bacteria on pathogen resistance, feeding efficiency, and growth in wild and aquaculture-reared fish (de Bruijn et al., 2018; Dulski et al., 2020; Parata et al., 2020; Bereded et al., 2021). Besides their role toward hosts, fish gut microbiomes are also considered as important reservoirs and dispersal agents of microbial diversity (Troussellier et al., 2017).

Owing to their diversity and multiple habitats and lifestyles, teleost fishes are excellent models to address the factors governing gut microbiome composition in vertebrates (Lescak and Milligan-Myhre, 2017; Mallott and Amato, 2021). Host phylogeny, leading to the discussed concept of phylosymbiosis (Brooks et al., 2016), and other factors including trophic level and diet, appear as important drivers of gut bacterial community composition (Ringø et al., 2016; Egerton et al., 2018). Besides host-related factors, many environmental factors including habitat, water quality, temperature, or captivity also contribute shaping gut communities (Sullam et al., 2012; de Bruijn et al., 2018; Kokou et al., 2019; Kim et al., 2021). Fewer studies have investigated seasonal effects [summarized in Egerton et al. (2018)]. Some of these found a strong influence of season-related variations in temperature and day length on gut microbiota of fish in aquaculture settings (Hovda et al., 2012; Neuman et al., 2016; de Bruijn et al., 2018). Captivity, either for aquaculture or in lab rearing facilities, is also well-known to influence gut community compositions in vertebrates and fish as a consequence of modifications in habitats and feeding habits (Roeselers et al., 2011; Hird, 2017; de Bruijn et al., 2018; Egerton et al., 2018; Alessandri et al., 2019). Increasing number of studies also emphasize the influence of exposure to human-introduced contaminants in microcosm experiments on model teleost species and support the emergence of a microbiome-aware ecotoxicology (reviewed in Evariste et al., 2019; Duperron et al., 2020). Naturally occurring compounds of different origins (e.g., produced by organisms, or released from land) may also have a large influence on the microbiome compositions. Among others, metabolites produced by the bloom-forming cyanobacterium *Microcystis aeruginosa* were shown to greatly modify the structure of gut communities in reared medaka fish (Duperron et al., 2019; Gallet et al., 2021).

Evaluating the relative importance of host versus environmental parameters in shaping fish gut communities requires comparing distinct species from a similar habitat. A limited number of studies have compared microbiomes of co-occurring fish species, even fewer that investigated several species from a single teleost family to evaluate the relative influence of phylogenetic versus environmental parameters (Sullam et al., 2015; Gallet et al., 2019; Sylvain et al., 2019; Ruiz-Rodríguez et al., 2020; Escalas et al., 2021). Using such an approach, distinct feeding regimes were for example shown to have more influence than phylogenetic history on the composition and relative abundances of various bacterial lineages in Sparidae (Escalas et al., 2021). Another factor that remains overlooked is the inter-individual variability, which can be high because individuals may correspond to diverse life stages, different physiological conditions, and of course experience random effects (Star et al., 2013; Stephens et al., 2016; Duperron et al., 2019; Ruiz-Rodríguez et al., 2020). Finally, most studies focus on fish species from temperate areas. Less is known about wild fish gut microbiome diversity in tropical areas, where most studies address skin and gut microbiome of coral reef fish (Chiarello et al., 2018; Cheutin et al., 2021). Data from wild fish species from freshwater water bodies, for example, include some works on cichlids from Lake Tanganyika, and crater lakes in Nicaragua (e.g., Baldo et al., 2019).

In this study, we investigated the effect of host versus environmental factors on the structure of gut-associated bacterial communities in four fish species collected from the Aghien lagoon (Ivory Coast). Aghien lagoon is a eutrophic freshwater lagoon and harbors high biomasses of microbial primary producers including potentially toxic cyanobacteria (Mitroi et al., 2020; Ahoutou et al., 2021). In the context of an international project addressing the sustainable monitoring of surface water resources used for the production of drinking water, specimens of commonly consumed fish species were collected over a year. These included the three Cichlidae *Hemichromis fasciatus*, *Tilapia guineensis* (syn. *Coptodon guineensis*), *Sarotherodon melanotheron* and the Claroteidae *Chrysichthys nigrodigitatus*. *T. guineensis* and *C. nigrodigitatus* were collected monthly, allowing exploring intra-annual variability of gut community compositions and its link with environmental parameters (water communities, rainfalls, turbidity…). A PCR-based 16S rRNA gene sequencing approach was employed to investigate the composition of bacterial communities. This study provides the first report on gut microbiota associated with fish displaying contrasting dietary behaviors in an eutrophicated freshwater ecosystems in West Africa.

## Materials and methods

### Study site and sampling

**FIGURE 1:**
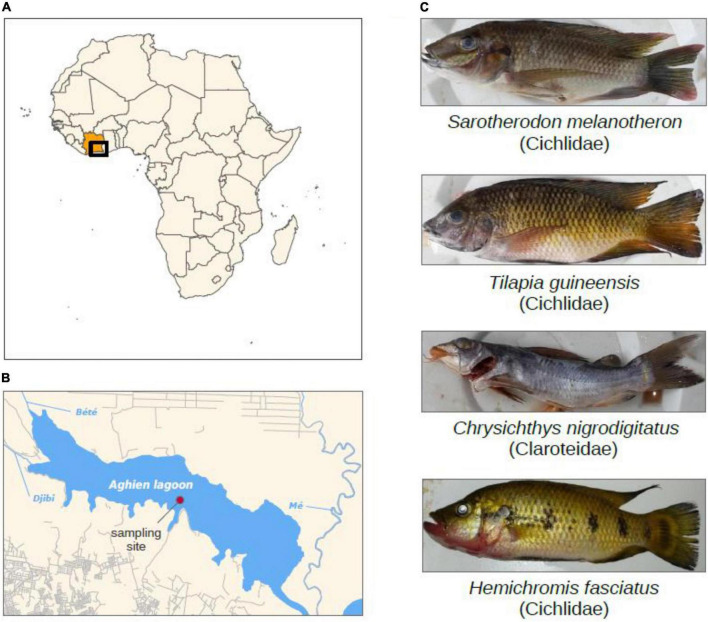
**(A,B)** Maps of the study area, the Aghien lagoon, located to the North-East of Abidjan in Ivory Coast (Africa) and water sampling site. **(C)** Four fish species and water were sampled monthly from the lagoon. Water sampling site is indicated.

Fish and water were sampled monthly between May 2017 and April 2018 in the Aghien lagoon (Ivory Coast) in the framework of the WaSAf project^1^ (Figures 1A,B). Aghien lagoon is a freshwater lagoon that covers a surface of 19.5 km^2^ for a perimeter of 40.72 km, a 25 km^3^ volume and a ∼10 m maximal depth (Koffi et al., 2019). It is part of a set of connected lagoons, east of the economic capital city Abidjan. The construction of a dam limits the effects of tides and the saltwater incursion in the lagoon (water salinity is below 0.03 per mil). Two rivers (Bété and Djibi) flow directly into the western part, and river Mé flows into the Aghien-Potou channel (Figure 1B). This lagoon is directly surrounded by several villages and ∼11,000 inhabitants (Rose et al., 2017). Local populations use the lagoon for domestic (washing, bathing, cooking, and waste disposal) as well as economics activities (fishing, agriculture) (Rose et al., 2017). Sampled fish belonged to four species commonly consumed, namely *H. fasciatus*, *T. guineensis*, *S. melanotheron* (three Cichlidae the former a carnivore, the other two being omnivores), and *C. nigrodigitatus* (Claroteidae, adults being carnivorous, mostly zooplanktonophagous). They were obtained from fishermen and measured (Figure 1C and Supplementary Table 1). Specimens of *T. guineensis* and *C. nigrodigitatus* were sampled throughout all eleven sampling events (29 and 31 specimens, respectively, Table 1). *S. melanotheron* was sampled only during the first four sessions (May–August 2017, 10 specimens), while *H. fasciatus* was sampled from November 2017 to February 2018 (11 specimens), these species not having been found at other dates. Gut was dissected immediately and frozen. As fish are mobile, water was sampled monthly from the central area of the lagoon. Water was sampled over the first meter depth using an integrative sampler (see Ahoutou et al., 2021), filtered (0.22 μm nitrocellulose filters, 11 samples, Table 1), and filters were frozen. Average values of various environmental data collected from six sampling stations along a west-east transect, are available in Ahoutou et al. (2021). Based on these previous results, turbidity, rainfall and Chlorophyll*-a* (Chl*a*) concentration were the most variable parameters and were thus used in this study. Notably, water temperatures over the sampling period oscillated between 26–31^°^C, and eutrophication levels were with high phytoplankton biomasses expressed as Chlorophyll*-a* (Chl *a*) concentration (26–187 μg.l^–1^ Chl*a*).

**TABLE 1:** Water and fish samples collected monthly in the Aghien lagoon over a year.

	2017− May	2017− Jun	2017− Jul	2017− Aug	2017− Oct	2017− Nov	2017− Dec	2018− Jan	2018− Feb	2018− Mar	2018− Apr	Total
*Sarotherodon melanotheron*	3	3	2	2	*NA*	*NA*	*NA*	*NA*	*NA*	*NA*	*NA*	10
*Tilapia guineensis*	3	3	2	3	2	3	1	3	3	3	3	29
*Chrysichthys nigrodigitatus*	3	3	2	3	3	3	2	3	3	3	3	31
*Hemichromis fasciatus*	*NA*	*NA*	*NA*	*NA*	*NA*	2	3	3	3	*NA*	*NA*	11
Water	1	1	1	1	1	1	1	1	1	1	1	11

NA, not available.

### DNA isolation and Illumina sequencing of the V4–V5 region of the bacterial 16S rRNA-encoding gene

DNA was extracted from lagoon water filters, and from homogenized fish gut tissue (whole length) crushed using mortar and pestle to avoid the effect of longitudinal heterogeneity. Because separation between gut resident and transient community cannot be achieved in a perfectly consistent manner among different fish species, we chose to analyze whole gut rather than mucosa alone (Gajardo et al., 2016). The ZymoBiomics DNA mini kit (Zymo Research, Irvine, CA, USA) was used according to manufacturer’s instructions, after an initial step of cell disruption using beads (6 × 1 min). A ∼400 bp fragment of the V4–V5 region of the bacterial 16S rRNA-encoding gene was amplified using primers 515F and 926R following published PCR protocol (Parada et al., 2016). PCR products were sequenced on an Illumina MiSeq platform (2 × 300 bp, paired-end sequencing, GenoToul, Toulouse, France) and paired-ends were assembled. Blank extractions and a community of known composition were used as internal controls for the sequencing procedure. Raw reads were deposited into the GENBANK Sequence Read Archive (SRA, Bioproject PRJNA758258) database under accession numbers SAMN20081138–SAMN20081229.

### Gene sequence analysis

Gene sequence datasets were analyzed using QIIME2 (Bolyen et al., 2019). Amplicon Sequence Variants (ASVs) were identified using DADA2 after quality filtering (using default parameters of the *qiime2 dada2 denoise-single* method) and chimera removal (Callahan et al., 2017). Taxonomic affiliations were obtained by the sklearn-based classifier (SILVA 132–99 release) (Quast et al., 2013). Sequences matching “Eukaryota,” “Archaea,” “Unassigned,” “Mitochondria,” and “Chloroplast” were discarded. Datasets were normalized by rarefaction to 4,133 reads, following recommendation by Weiss et al. (2017), while two *T. guineensis* and one *S. melanotheron* samples with lower counts were discarded.

### Comparison of bacterial communities

Diversity indices (ASV richness, evenness) were calculated for water and fish gut samples using the package *vegan* (Oksanen et al., 2020) in R 4.1.0 (R Core Team, 2021). The comparison of species richness among the five sample types was performed with a Kruskal–Wallis rank sum test and pairwise comparisons, using the package *rstatix* (Kassambara, 2021). Bar plots were created using the packages *ggplot2* (Wickham, 2016) and *microshades* (Dahl et al., 2022). Principal coordinates analysis (unweighted and weighted UniFrac distances), PERMANOVA, pairwise comparisons, and bar charts were produced. Packages *phyloseq* and *RVAideMemoire* were employed (McMurdie and Holmes, 2013; Hervé, 2021). PERMANOVA comparisons involving *S. melanotheron* and *H. fasciatus* were conducted versus water, *T. guineensis* and *C. nigrodigitatus* samples collected from the same months only. Levels of variance within each sample type were analyzed performing PERMDISP using *vegan*. Venn diagrams were produced using the package *VennDiagram* (Chen, 2018).

## Results

### Diversity of gut bacterial communities

A total of 2,397,139 sequences were obtained following taxa filtering. Rarefaction to 4,133 reads per sample allowed curves to reach saturation (not shown), suggesting that this number was sufficient to gain a representative overview of bacterial diversity. Retained reads following rarefaction (380,236 reads) clustered into 12,313 ASVs, of which 1,265 ASVs (amounting to 73.5% of reads) represented at least 1% of reads in at least one sample (later on qualified as “abundant” ASVs). Only nine ASVs (3.5% of reads) represented at least 10% of reads in at least one sample (later on qualified as “dominant” ASVs).

**FIGURE 2:**
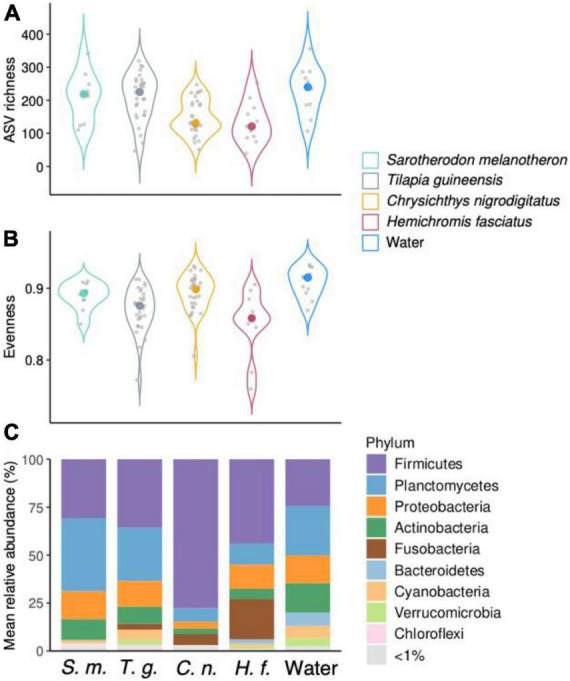
**(A,B)** Amplicon Sequence Variants (ASV) richness and evenness indices of bacterial communities from water and fish gut samples over all sampled months. Colored dots represent median values. **(C)** Mean relative abundance of the bacterial taxa at the phylum level across water and fish guts over all samples. Initials under the *x* axis represent the different sample types (genus species, water).

Levels of Amplicon Sequence Variants richness were higher in water (232 ± 72) compared to fish guts. Gut communities were in average more diverse in *T. guineensis* and *S. melanotheron* (209 ± 71 and 210 ± 73, respectively) compared to *C. nigrodigitatus* and *H. fasciatus* (148 ± 52 and 132 ± 63, respectively, Figure 2A). Significant differences in ASV richness existed among the five groups (Kruskal–Wallis rank sum test, *p* < 0.001), especially between *T. guineensis* and *C. nigrodigitatus* (Bonferroni-adjusted *p* = 0.004), and between water and the two fish *C. nigrodigitatus* and *H. fasciatus* (Bonferroni-adjusted *p* = 0.016 and *p* = 0.043, respectively). Evenness was high in all samples types, and comparable between water (0.91 ± 0.02) and fish guts (0.88 ± 0.03, Figure 2B). Firmicutes, Planctomycetes, Proteobacteria, Actinobacteria, and Fusobacteria were the most abundant phyla in all fish gut samples (Figure 2C). Water communities were also dominated by these four phyla (Planctomycetes, 26 ± 15%; Firmicutes, 25 ± 22%; Actinobacteria, 16 ± 6%; Proteobacteria, 14 ± 7%). Firmicutes were particularly abundant and dominant in *C. nigrodigitatus* (78 ± 26%, Figure 2C) while they never represented more than 44% in the other fish species. *H. fasciatus* gut samples were the only ones to present abundant Fusobacteria (21 ± 16%). Fusobacteria never represented more than 5.7% of reads in the other fish species.

### Annual dynamics of bacterial communities in water and in guts of *Tilapia guineensis* and *Chrysichthys nigrodigitatus*

**FIGURE 3:**
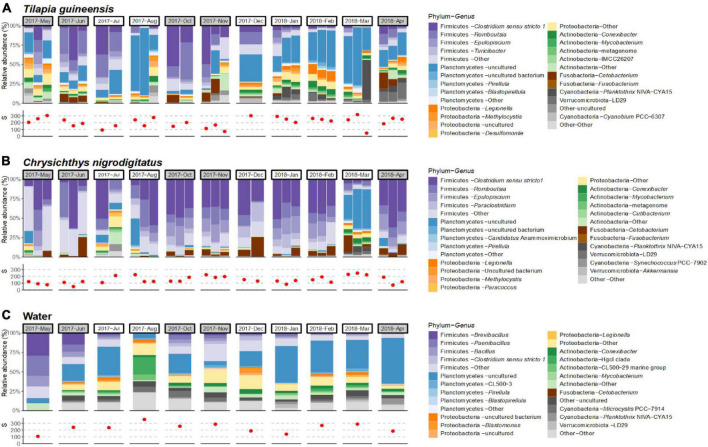
Temporal profiles over a year of community compositions for each individual sample at the genus level (only the most abundant genera within the overall dataset are displayed) and species richness (S), in *Tilapia guineensis* **(A)**, *Chrysichthys nigrodigitatus* **(B)**, and water samples **(C)**. Months corresponding to rainy seasons are colored in gray. Red dots represent observed amplicon sequence variant (ASV) richness. Affiliation “Other” corresponds to the group of low-abundance ASVs.

*Tilapia guineensis*, *Chrysichthys nigrodigitatus* and water were collected at every 11 sampling dates throughout the year, allowing to document the temporal variability of their bacterial community composition (Figures 3A–C). Large abundance variations of certain bacterial taxa were observed over the year, but the limited number of replicate specimens per date prevented robust statistical analysis.

The gut communities of *T. guineensis* showed consistent dominance of some phyla (Firmicutes, Planctomycetes, and Proteobacteria), with shifts in abundances (Figure 3A). Firmicutes were most abundant in June–July and October–November, with important contribution of genera *Clostridium sensu stricto* 1 and *Romboutsia*. Interestingly, these periods correspond to the two rainy seasons (April–June and October–November), when rainfalls and turbidity are higher, which are separated by two dry periods (July–September and December–March). Planctomycetes were abundant throughout the year except in October–November, notably the genus Planctomycetes – *uncultured* (5.5 ± 8.8% in October–November, versus 21.6 ± 19% at others dates) (Figure 3A). Qualitatively, relative abundances of Firmicutes increased and abundances of Planctomycetes decreased with increased rainfalls and, to a lesser extent, turbidity (Supplementary Figure 1).

*Chrysichthys nigrodigitatus* communities on the other hand appeared relatively stable at the phylum level, except in July 2017 (for one fish) and in March 2018 when more heterogenous and diverse communities were observed (Figure 3B). No self-evident trend in Firmicutes or Planctomycetes abundance was observed with either rainfalls, turbidity, or Chl*a* content (Supplementary Figure 1). Overall, communities were dominated by Firmicutes except in the three specimens sampled in March 2018 in which they represented 15 ± 4% compared to mean 84 ± 16% for other sampling dates. At this date, Planctomycetes were particularly abundant (43 ± 11%). At the genus level, *Clostridium sensu stricto* 1 was the main Firmicutes throughout the sampling year (27 ± 17%, Supplementary Figure 2). Interestingly, shifts were observed within the Firmicutes, including genera *Turicibacter* and *Epulopiscium* (Supplementary Figure 2). *Turicibacter* was abundant in specimens sampled in May–June (20 ± 18%), and low in other sampling events (1.3 ± 1.1%). Genus *Epulopiscium* (Lachnospiraceae) was totally absent in June–July, December, and March, while abundant in other sampled months (15 ± 4%).

Water communities were visually stable over the year at the phylum level, except noticeable variations in May and August. Firmicutes were highly dominant in May (80%) compared to other months (21 ± 11%), while absent in August (Figure 3C). ASV richness and percentage of dominant taxa (Firmicutes, Planctomycetes, and *Clostridium sensu stricto* 1) in water, *T. guineensis* and *C. nigrodigitatus* samples, were compared to published values for rainfalls, turbidity and Chl*a* concentrations (Ahoutou et al., 2021). No self-evident trend was observed between richness or percentage and any of these variables (Supplementary Figure 1).

### Comparison of community compositions in water and fishes

**FIGURE 4:**
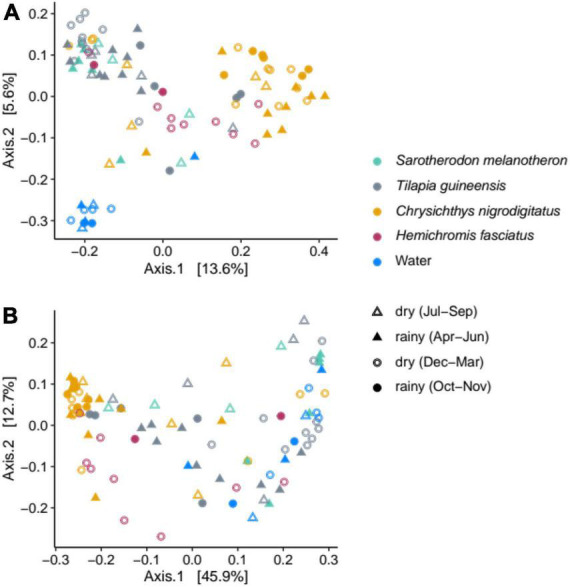
Principal coordinates analyses based on the unweighted **(A)** and weighted **(B)** UniFrac distances, illustrating the bacterial compositions from water and fish gut samples according to the different seasons over the year (dry or rainy).

Community compositions from water samples and from the four fish species were compared. Water samples were well separated from all fish gut communities on the principal coordinates analysis (PCoA) based on unweighted UniFrac distance, suggesting differences in terms of bacterial membership (Figure 4A), while separation was less obvious on the PCoA when also considering abundances (weighted UniFrac distance, Figure 4B). Pairwise comparisons over 11 months revealed significant differences among water, *T. guineensis* and *C. nigrodigitatus* based on both weighted and unweighted UniFrac (*p* = 0.001). Because *S. melanotheron* and *H. fasciatus* were each sampled during 4 months only, comparisons were conducted including only May–August for water and other species for the former, and November–February for the latter (Table 1). They are thus to be taken with caution given that each of these two species was only sampled during 4 months, with no overlap between the two sampling periods. PERMANOVAs were significant for all comparisons except between *S. melanotheron* and *T. guineensis* (pairwise comparison PERMANOVA, Bonferroni adjusted, *p* > 0.27). Levels of intra-group variance were not different (PERMDISP, weighted UniFrac, *p* = 0.3), suggesting that the detected differences in community compositions are statistically robust even for the shorter sampling periods.

**FIGURE 5:**
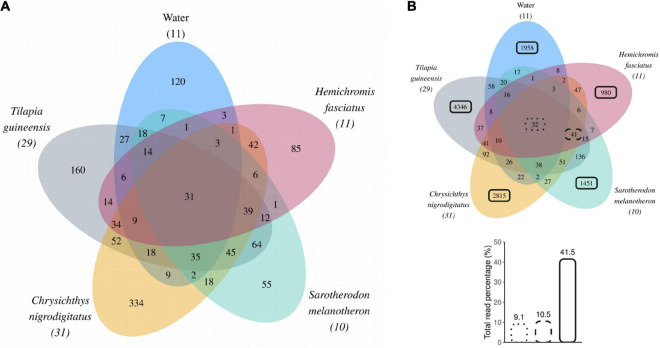
**(A)** Venn diagram displaying Amplicon Sequence Variants (ASVs) abundant in at least one sample across water or fish guts. Numbers in brackets represent numbers of samples collected over the year for each sample type. **(B)** Venn diagram including all ASVs shared between water or fish gut samples. Black boxes (dotted, dashed, or solid lines) highlight ASVs for which the reads contribution was calculated and expressed as a percentage of total reads.

Thirty-two Amplicon Sequence Variants were common to all fish species as well as water (representing 9.1% of total reads, Figure 5). These include ASVs affiliated to bacterial genus *Romboutsia* (4 ASVs), genera from families Clostridiaceae 1 (12 ASVs, mostly genus *Clostridium sensu stricto 1*), and Pirellulaceae (10 ASVs); and to cyanobacteria like genus *Planktothrix* (1 ASV). According to BLAST_*N*_ results, *Romboutsia* ASVs may come from environmental or animal-associated gut bacteria. The *Planktothrix* ASV best matched with a *Planktothrix tepida* strain isolated from the Aghien lagoon. Clostridiaceae 1 ASVs matched with freshwater habitat-associated bacteria identified during cyanobacterial blooms, or with fish gastrointestinal microbial communities (yellow catfish, Nile Tilapia). Pirellulaceae ASVs were mainly associated with bacterioplankton from eutrophic or cyanobacterial bloom-associated freshwater lakes, whose bacteria probably came from the cyanobacteria mucilage. Thirty-one of the 32 ASVs shared between fish species and water were abundant (i.e., > 1% of reads in at least one sample) and overall represented 9.0% of the total reads (Figure 5A). Additional 41 ASVs (of which 39 abundant), representing 10.5% of total reads, were shared by all fish species but absent in water (Figure 5B) including 25 belonging to genera commonly described from gut communities, namely *Clostridium sensu stricto* 1 (10 ASVs), *Romboutsia* (6 ASVs), *Cetobacterium* (5 ASVs), *Paraclostridium* (3 ASVs), and *Turicibacter* (1 ASV). According to BLASTN results, *Clostridium sensu stricto 1* ASVs best matched with bacteria from freshwater habitats or from fish gut communities. *Romboutsia* ASVs were most similar to gut bacteria. *Cetobacterium* ASVs matched with bacteria from gastrointestinal tract of various fish including cichlids. *Paraclostridium* ASVs matched with bacteria from fish gastrointestinal tract community or environment. Finally, the *Turicibacter* ASV matched with gut and environmental bacteria.

### Diversity of non-shared Amplicon Sequence Variants

A total of 1,958 ASVs (of which 120 were abundant) were unique to water, while fish species displayed between 980 and 4,349 unique ASVs with 55–334 considered as abundant (Figures 5A,B). These ASVs unique to a single sample type overall accounted for 93.8% (11,550 ASVs) of all identified ASVs and represented 41.5% of the total reads (Figure 5B). Among these non-shared ASVs, 93.5% (10,796 ASVs) were rare (i.e., never represented above 1% of reads in any sample). This indicates that most of the bacterial diversity found in this study consists of rare ASVs that were found in a single sample type. Specifically, 77.9% of identified ASVs were found unique to a single of the four fish species and 15.9% were found only in water.

## Discussion

### Fish and water harbor distinct associated bacterial communities

This study contributes new data to the poorly studied fish-associated gut bacterial communities in the African waters (Elsaied et al., 2021). As expected based on previous fish microbiome studies from diverse habitats, gut-associated communities were different from those observed in the water, although some ASVs were shared, the latter possibly corresponding to transient bacteria (Härer et al., 2020; Gallet et al., 2021). The dominant phyla found in fish guts, namely Firmicutes, Proteobacteria and Fusobacteria, are commonly encountered in freshwater fish (Miyake et al., 2015; Liu et al., 2016; Härer et al., 2020; Riera and Baldo, 2020; Kim et al., 2021; Park and Kim, 2021). They include members of the cichlid core microbiota, as three of the species investigated here belong to this family (*S. melanotheron*, *T. guineensis*, and *H. fasciatus*) (Baldo et al., 2015, 2019). Indeed, most of the abundant ASVs correspond to genera commonly reported as gut-associated symbionts (for example *Turicibacter*) and they include *Clostridium sensu stricto* 1 and *Cetobacterium* which are almost ubiquitous in cichlids (Kamada et al., 2013; Baldo et al., 2019; Riera and Baldo, 2020). Presence of *Epulopiscium* (Firmicutes) is interesting as this is one of the largest bacteria, identified in the gut of surgeonfish where it participates digestive pH regulation and may assist food processing in herbivorous species (Miyake et al., 2016). It contains multiple copies (up to 100,000) of its genome, which is a reminder that relative abundances estimated based on read abundances may not reflect actual bacterial abundances (Amend et al., 2010). *Chrysichthys nigrodigitatus* harbors a higher proportion of Firmicutes compared to the three cichlid species. Firmicutes are well known to numerically dominate in various vertebrate gut communities (Ley et al., 2008b). *Clostridium sensu stricto* 1 (cluster I) was the major Firmicutes genus observed within fish samples, previously reported to be particularly abundant in herbivorous fish species and which includes saccharolytic and proteolytic species (Collins et al., 1994; Liu et al., 2016; Li et al., 2017). Higher relative abundances of Fusobacteria in the carnivorous species *Hemichromis fasciatus* are consistent with previous findings supporting their significance in carnivores (Liu et al., 2016). Fusobacteria mostly consisted of *Cetobacterium* in this study, a genus described as an obligate gut associate, well known to contribute to fish health by producing B_12_ vitamins (Tsuchiya et al., 2008; Gallet et al., 2021). Planctomycetes, the second most dominant phylum in fish samples as well as water, includes both environmental as well as host-associated bacteria, and it is thus unclear whether it consists mostly of resident or transient members of the gut microbiota, yet Planctomycetes were previously identified in guts of numerous cichlids species from lakes in Africa (lakes Barombi Mbo and Tanganyika) and Nicaragua (van Kessel et al., 2011; Baldo et al., 2019; Elsaied et al., 2019; Riera and Baldo, 2020). The majority of Planctomycetes were affiliated to Pirellulaceae, with a few identified as *Pirellula*. According to Clum et al. (2009), *Pirellula* strains are able to attach to cyanobacteria, suggesting that some could originate from the phycosphere and be ingested along with the phytoplankton (Bi et al., 2019). Indeed, Aghien lagoon is experiencing frequent cyanobacterial bloom events (Mitroi et al., 2020). The toxin-producing cyanobacterial genus *Planktothrix*, recently documented from the cyanobacterial phytoplankton community in Aghien lagoon (Ahoutou et al., 2021) was for example found both in water (yet not dominant) as well as occasionally in fish guts.

### Fish guts are reservoirs of rare bacteria in the Aghien lagoon

Several recent studies have emphasized the importance of animals as reservoirs and dispersal agents for rare bacteria. In a recent census of the literature, macro-organism-associated bacteria were found to represent a significant fraction of the total bacterial diversity in the marine realm (Troussellier et al., 2017). Another study found that 90% of the prokaryotic diversity associated with metazoans tissues was not recovered in the surrounding plankton on a coral reef around the Mayotte island, and extrapolated that coral reef animal-associated microbiota could represent up to 2.5% of the total prokaryotic diversity on Earth (Chiarello et al., 2020). A similar trend is observed in our dataset. Indeed, the vast majority (77.9%) of all identified ASVs were found only in one of the four fish species considered. Each individual fish species harbored a number of non-shared ASVs similar to the number found in water (average 2,398 vs. 1,958), supporting that fish are also important reservoirs of bacterial diversity in the Aghien lagoon. Similar to the aforementioned studies, this diversity mostly consists of rare bacteria. It is not yet clear how significant this diversity is to overall ecosystem functioning, resistance, or resilience, but studies have shown that some of these rare bacteria can thrive under particular and unusual conditions and then become abundant, ultimately contributing significantly to ecosystem functioning (Aanderud et al., 2015). It further emphasizes the relevance of conservation policies that help maintain fish diversity and its underlying important bacterial diversity in anthropized freshwater ecosystems.

### *Tilapia guineensis* and *Chrysichthys nigrodigitatus* gut bacterial communities vary over a year

Time-series monitoring of gut community compositions in diverse wild species are of increasing interest to better understand the contribution of environmental factors to community structure, but remain rare in the literature, in particular for wild fish (Al-Harbi and Naim Uddin, 2004; Dulski et al., 2020; Le et al., 2020). Results from *T. guineensis* and *C. nigrodigitatus* revealed high levels of intra-annual variability in gut bacterial communities over the 11 months of sampling. We attempted to relate those variations to the most variable physico-chemical parameters measured in the Aghien lagoon over this period (Ahoutou et al., 2021). Notably, two rainfall peaks occurred in June and October 2017 and turbidity was highly variable with peaks in April–June, and October 2017. Indeed, rainfalls were recently identified as main drivers of physico-chemical and plankton characteristics of the Aghien lagoon ecosystem, being positively correlated with P, N and turbidity, and negatively with temperature and phytoplankton biovolumes (Ahoutou et al., 2021), with no clear seasonality. Interestingly, gut communities from *T. guineensis* displayed variations in phyla abundances, such as higher relative abundance of Firmicutes and lower abundances of Planctomycetes when rainfalls and turbidity increased (the latter two being correlated). Dominance of Firmicutes, mainly members of the *Clostridium sensu stricto* 1 and *Romboutsia* genera, in June–July and October–November, correspond to higher rainfall and turbidity periods. *Clostridium sensu stricto* 1 and *Romboutsia* are reportedly abundant genera in tilapia fish, for example in both wild and aquaculture-reared Nile tilapia specimens (genus *Oreochromis*; Bereded et al., 2021, 2022). They are also abundant in the gut of many vertebrates and often reported in wastewater (Boukerb et al., 2021). *Clostridium sensu stricto* was for example reported to be primarily transported to surface waters by runoff events after manure application (Rieke et al., 2018). Bacterial genus *Romboutsia* comprises flexible anaerobes that are well-adapted to the intestine environment, and produce straight-chain fatty acids that are likely significant to host health (Gerritsen et al., 2017; Wu et al., 2021). Yet, both genera can also be abundant in lake water as observed recently in the thalassohaline lake Tuz in Turkey, where *Romboutsia* and *Clostridium sensu stricto* 1 represented above 35 and 8% of 16S rRNA sequences, respectively (Oyewusi et al., 2021). Their increase in fish guts during high rainfall and turbidity episodes in the Aghien lagoon could thus be linked to increased runoffs, that may transport bacteria to the lagoon or promote enrichment in organic matter that may locally favor their growth. These events may modify the trophic regime of fish with possible impact on fish gut microbiota. On the other hand, variations in phytoplankton biomass (as estimated by Chl*a* content) do not appear to be directly related to gut community compositions.

At this stage, the limited number of replicate samples does not allow to robustly test the relationships between microbiota structure and environmental parameters. Yet, the mere fact that some important temporal variations are observed in *T. guineensis*, less so in *C. nigrodigitatus*, points to the relevance of time-series sampling for proper evaluation of microbiome composition, and to the existence of potential species-related differences in this temporal variability.

### Host phylogeny, diet, and composition of fish gut bacterial communities

In spite of large variation of compositions over time, communities from *T. guineensis* and *C. nigrodigitatus* separate clearly from each other, from water, and from *H. fasciatus*. This indicates that host species-specific effects on gut community compositions exceed intra-specific temporal variability. Communities in *T. guineensis* and *S. melanotheron* on the other hand appear similar despite only ten specimens of the latter were available, collected over only 4 months. Both these species belong to the Pseudocrenilabridae subfamily (Cichlidae), but so does *H. fasciatus*. The omnivorous feeding regimes of the former two are broad, and comprise periphyton as well as plankton and animal-derived material (Froese and Pauly, 2021), while adult *H. fasciatus* is a strict carnivore (Assi et al., 2019; Imorou et al., 2019). In the Cichlidae family, transition between carnivory to herbivory was shown to be a major event influencing gut community compositions (Baldo et al., 2017). Specialized carnivores display lower alpha diversity compared to herbivores, correlated with shorter digestive tract and transit time (Ley et al., 2008b). *H. fasciatus* gut communities observed in this study fit with this hypothesis, as they display lower species richness as well as distinct community compositions compared to the two other cichlids, including a higher abundance of Fusobacteria which is commonly reported in carnivores (Liu et al., 2016). This supports that the differences observed here among cichlids are likely due to their distinct diet habits. *C. nigrodigitatus* belongs to a different family (Claroteidae), and is omnivorous as a juvenile, with a trend toward carnivory with increasing age and size (Froese and Pauly, 2021). Interestingly, it also displays lower microbiome species diversity compared to *T. guineensis* and *S. melanotheron*. This is consistent with the hypothesis that carnivory is generally associated with lower gut bacterial diversity compared to herbivory and omnivory. This trend is also demonstrated in mammals, and is hypothesized to be related to the shorter length of the tract and transit duration in carnivores, which limits the possibility of microniche differentiation for example with regards to oxygen availability (Ley et al., 2008a; Nishida and Ochman, 2018). However, this trend may not be generalized to all teleost fish because of potential flexible diets and/or opportunistic consumptions following resources variations (Liu et al., 2016). Further disentangling the respective influence of phylogeny or ontogeny versus feeding regime on gut-associated community compositions in Aghien lagoon fish will require extending the number of species and specimens analyzed, in particular by including several other carnivorous cichlids in the present case (Sullam et al., 2012), and ascertaining the exact feeding regime of collected specimens, for example by analyzing gut contents and stable isotope compositions.

## Conclusion

Distinct fish species co-occurring in the Aghien lagoon harbor distinct bacterial communities that are influenced by host phylogeny as well as diet. Community composition may vary with time in a given fish species. This work emphasizes the challenges in disentangling the respective role of host and environmental factors in shaping gut communities, because of the complexity of natural settings and the multiplicity of confounding factors (correlated variables, limited availability of certain species). Further work is now needed to properly identify the causal factors (intrinsic and extrinsic) that influence fish microbiome and health in the lagoon, and their long-term effects on fish populations sustainability. To do so, reductionist approaches under controlled conditions are necessary, alongside holistic approaches using more environmental samples. These may for example take advantage of the existence of aquaculture settings in the Aghien lagoon, or may involve field-based mesocosm experiments (Ahoutou et al., 2022).

## Data availability statement

The datasets presented in this study can be found in online repositories. The names of the repository/repositories and accession number(s) can be found in the article/Supplementary material.

## Ethics statement

Ethical review and approval was not required for the animal study because fish used in this study were initially collected by fishermen for sale; no experiment was conducted on live specimens.

## Author contributions

CB, CQ, J-FH, JC, MT, and SD conceived the project and the study. CB, EY, CQ, J-FH, JC, and MT collected and prepared the samples. AG, EY, PF, BM, and SD performed the lab experiments, generated, and analyzed the data. AG, PF, and SD wrote the manuscript. All authors edited and agreed on the submitted version of the manuscript.

## Footnotes

1
https://humbert19.wixsite.com/wasaf
