# Supplementary material for: Fish gut-associated bacterial communities in a tropical lagoon (Aghien lagoon, Ivory Coast)
Source: Front Microbiol. 2022 Sep 29;13:963456. doi: 10.3389/fmicb.2022.963456 (PMC9556852; doi:10.3389/fmicb.2022.963456)
Supplement: Supplementary file 1 [file Data_Sheet_1.zip › Supplementary Figures.DOCX]

**Supplementary figure 1:** Scatter plots representing median values of ASV richness values for each sample type **(a-c)**, Firmicutes abundances **(d-f)**, Planctomycetes abundances **(g-i)** and *Clostridium sensu stricto 1* abundances **(j-l)** according to rainfall, turbidity or Chlorophyll *a* concentrations values over the sampling year, as reported in Ahoutou et al, 2021. Due to low replicate numbers, no statistical testing was conducted and only trends are presented.


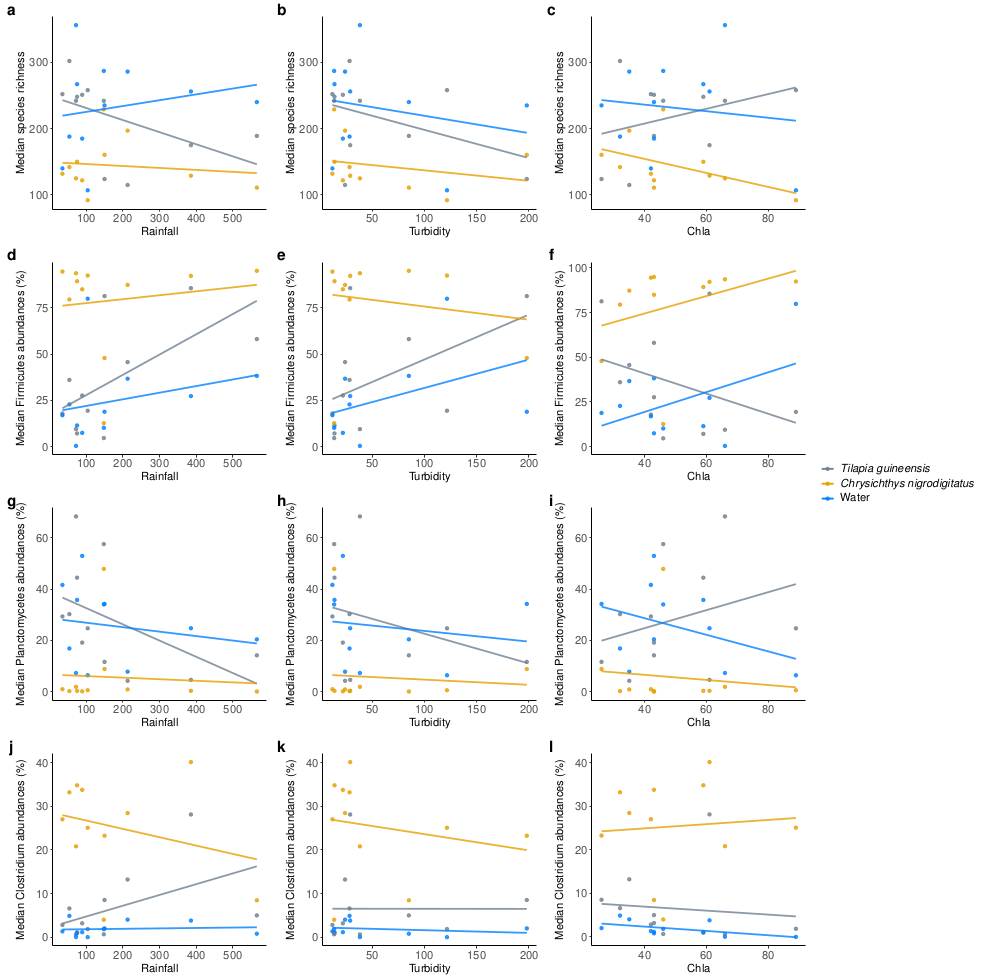


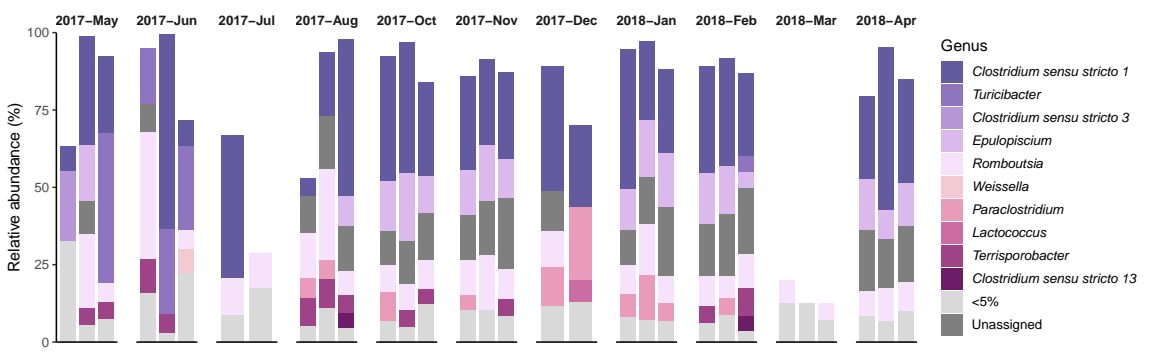
**Supplementary figure 2:** Firmicutes relative abundances at the genus level in *Chrysichthys nigrodigitatus* individuals sampled over a year. Genera with low abundances, i.e. under 5% in each individual, were pooled together and labeled « <5% ».
